# Breast Cancer Awareness and Barriers to Early Presentation in the Gaza-Strip: A Cross-Sectional Study

**DOI:** 10.1200/JGO.18.00095

**Published:** 2018-10-29

**Authors:** Mohamedraed Elshami, Hanan Abu Kmeil, Maymona Abu-Jazar, Ibtisam Mahfouz, Dina Ashour, Ansam Aljamal, Nada Mohareb, Reem Elbalaawi, Reem Dabbour, Jomana Ghaith, Tayseer Hasan, Meral Abdelati, Esraa Saleh, Haifa Shawwa, Reem Al-Ghazali, Ola Obaid, Loai Albarqouni, Bettina Böttcher

**Affiliations:** **Mohamedraed Elshami**, **Reem Dabbour**, **Tayseer Hasan**, **Esraa Saleh**, and **Haifa Shawwa**, Ministry of Health; **Hanan Abu Kmeil**, **Maymona Abu-Jazar**, **Ibtisam Mahfouz**, **Dina Ashour**, **Ansam Aljamal**, **Nada Mohareb**, **Reem Elbalaawi**, **Meral Abdelati**, **Reem Al-Ghazali**, and **Bettina Böttcher**, Islamic University of Gaza School of Medicine; **Jomana Ghaith**, Alazhar University School of Medicine, Gaza, Palestine; and **Loai Albarqouni**, Centre for Research in Evidence-Based Practice, Bond University, Australia.

## Abstract

**Purpose:**

Timely detection of breast cancer (BC) is important to reduce its related deaths. Hence, high awareness of its symptoms and risk factors is required. This study aimed to assess the awareness level of BC among females in Gaza.

**Materials and Methods:**

A cross-sectional study was performed during September and October 2017 in Gaza, Palestine. Stratified sampling was used to recruit patients from four hospitals and seven high schools. The validated Breast Cancer Awareness Measure (BCAM) was used to assess confidence and behavior in relation to breast changes, awareness of BC symptoms and risk factors, barriers to seek medical help, and knowledge of BC screening. Women (age ≥ 18 years) visiting or admitted to any of the four hospitals, and female adolescents (age 15 to 17 years) in any of the seven schools, were recruited for face-to-face interviews to complete the BCAM.

**Results:**

Of 3,055 women approached, 2,774 participants completed the BCAM questionnaire (response rate, 90.8%); 1,588 (57.2%) were adults, and 1,186 (42.8%) were adolescents. Of these, 1,781 (64.2%) rarely (or never) checked their breasts, and 909 (32.8%) were not confident to notice changes. In total, 1,675 (60.4%) were aware of the availability of BC screening programs. The overall mean ± standard deviation score for awareness of BC symptoms was 5.9 ± 2.9 of 11, and that of risk factors 7.5 ± 3.1 of 16. Feeling scared was the most reported barrier to seeking advice reported among women (n = 802; 50.2%), whereas feeling embarrassed was the most reported in adolescents (n = 745; 62.8%).

**Conclusion:**

Awareness of BC symptoms, risk factors, and screening programs is suboptimal in Gaza. Educational interventions are necessary to increase public awareness of BC and to train local female breast surgeons to address barriers to early detection.

## INTRODUCTION

Breast cancer (BC) is the most commonly diagnosed cancer among women worldwide.^1^ In Gaza, BC accounts for 31.3% of all reported cancer occurrences among Palestinian women: its prevalence is 149.1 per 100.000. In Palestine, BC is the leading cause of cancer-related deaths among women: its mortality rate is 9.8%.^2^

This could be attributed to the diagnosis at advanced stages because of low awareness levels of BC symptoms or risk factors and difficult access to health care. Hence, the importance of knowledge about BC signs and symptoms as well as risk factors (eg, age, family history, early menarche, and hormone replacement therapy^3,4^) and the need for every woman to be aware of and engaged with a BC screening cannot be under-emphasized.

Additional reasons for the relatively high prevalence of BC in Palestine include the presence of social stigma, fear, and reduced engagement in screening behaviors, such as breast self-examination (BSE).^5^ BSE is one of the three screening tests considered for early detection of BC: clinical breast examination, x-ray mammography, and BSE.^6^ In 2003, the American Cancer Society recommended education about BSE for women age 20 years and older, but women should know that BSE is most suitable for interval screening.^7^ It is important to realize that the concepts of breast awareness and BC awareness are inextricably linked, because women must be confident to look at and feel their breasts so that they can know what is normal for their own body and what changes to notice and then report to their health care providers.^8-10^

The poor knowledge and misleading beliefs about BC among women are responsible for a negative perception of the likelihood for a cure if detected early and the efficacy of screening tests.^11^ Studies that tackled the different aspects of BC awareness among Arab women were few and revealed a lack of BC knowledge among women.^12-15^ In Gaza, breast screening is available, but the program is not disseminated well, and no general awareness of such a program exists. Only women who get to know about the program and the location of screening from a health care professional usually can take part—many women remain unaware of the available screening program.^16,17^

To our knowledge, this is the first study conducted in the Gaza Strip to assess BC knowledge among women in Gaza, the attitude of these women toward BC, the awareness of BC age-related and lifetime risks and availability of a BC screening program, and to uncover the barriers to seeking medical help.

## MATERIALS AND METHODS

### Study Design and Population

A cross-sectional study was conducted from September 1 to October 31, 2017. It assessed BC awareness and attitudes toward BC among adolescent and adult women in the Gaza Strip. As an assessment tool, the Breast Cancer Awareness Measure (BCAM) questionnaire, which is a validated standardized measurement for BC awareness in the general population, was used.^18^ The questionnaire consists of three sections; the first evaluated behaviors in relation to breast changes as well as knowledge of age-related and lifetime BC risk in addition to the availability of a BC screening program in Gaza. The second section posed open-ended (recall) questions, and the third included closed (recognition) questions with a comparison between both outcomes (recall *v* recognition).

A 3-point Likert scale was used to evaluate knowledge of BC symptoms and signs as well as to explore barriers to seeking medical help, which were categorized into emotional, practical, and service barriers. A 5-point Likert scale was used to assess the awareness of BC risk factors.

The BCAM was translated from English to Arabic and then back-translated into English by several people proficient in both languages. A pilot study was conducted with 78 respondents to test the clarity of the questions of the Arabic BCAM version. A reliability analysis was carried out on the resulting task value scale composed of 29 items. Cronbach’s α showed that the questionnaire reached acceptable reliability (α = .757). Most items were worthy of retention; that is, deletion of the item resulted in a decrease in the α.

### Sampling Methods

Governmental hospitals are the main entry point for health care services in Gaza. Therefore, adult women age 18 years or older who were admitted to or visiting those hospitals were the target population and were recruited to participate. Patients or visitors to oncology departments were excluded from the study.

There are 13 hospitals in the Gaza Strip.^2^ From these, the largest four hospitals, located in four separate geographic locations in the Gaza Strip—namely, the North, including Gaza City; the Middle Governorate, Khan Younis, and the South—were chosen for recruitment of adult women and adolescent participants by stratified sampling. This sampling area covered most of the Gaza population and produced a representative sample from across the Gaza Strip. Parallel to this, adolescents from seven high schools (of 75 female-only high schools in Gaza^19^) located in the same areas as the study hospitals were recruited to achieve uniformity of areas. Adolescents age 15 to 17 years study health-related topics in their high-school curriculums, so there is an opportunity to explore their awareness of BC. Participants were invited to face-to-face interviews for completion of the BCAM.

Data collectors were trained to recruit participants, distribute questionnaires, and facilitate completion. They were also trained to administer the questionnaire to illiterate participants. Before completion of the questionnaire, a detailed explanation of the study, including its purposes, was given to the participants. Informed consent was obtained from the participants, and ethical approval was obtained from both the Palestinian Ministry of Health and the Ministry of Higher Education.

### Statistical Methods

Descriptive statistics were calculated for demographic data and behaviors in relation to breast changes by the participants. Cumulative scores were then calculated for the recognition of BC signs and symptoms, risk factors, and reported barriers. When recognition questions of BC symptoms were used, the “I do not know” responses were considered ”No” responses; thus the 3-point Likert scale (ie, yes, no, I do not know) was converted into a 2-point scale (ie, yes or no) to facilitate comparison of different responses to recognition versus recall questions.^13^

The 5-point Likert scale was also converted into a 3-point scale, because it was difficult for participants to distinguish between the “agree” versus “strongly agree” and the “disagree” versus “strongly disagree” responses. The responses of “strongly agree” and “strongly disagree” were recoded to “agree” and “disagree,” respectively, using statistical software as described previously.^13^

The χ^2^ test was used to compare the awareness of each BC symptom between adults and adolescents. The variable of interest was the mean score of overall awareness, for which the one-sample *t* test was used for each section and the two-sample *t* test was used to compare the mean scores of knowledge among adults and adolescents. Data were analyzed using the Statistical Package for the Social Sciences version 22 (IBM Corporation, Chicago, IL).

## RESULTS

Of the 3,055 invited participants, 2,774 completed the BCAM questionnaire (response rate, 90.8%). Among them, 1,588 (57.2%) were adults and had a mean (standard deviation [SD]) age of 34.1 ± 12.2 years; 1,186 (42.8%) were adolescents and had a mean ± SD age of 16.2 ± 0.9 years (Table 1).

**Table 1:**
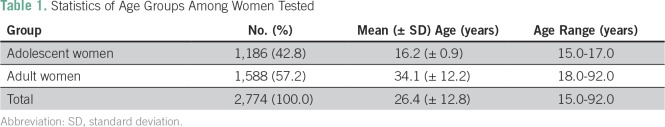
Statistics of Age Groups Among Women Tested

A total of 1,781 (64.2%) reported that they rarely (or never) checked their breasts, and 909 (32.8%) were not confident to notice changes (Table 2). Only 60 participants (2.2%) gave a correct answer for the age-related risk of BC, whereas 1,181 (42.6%) answered the question for lifetime risk correctly (Table 3).

**Table 2:**
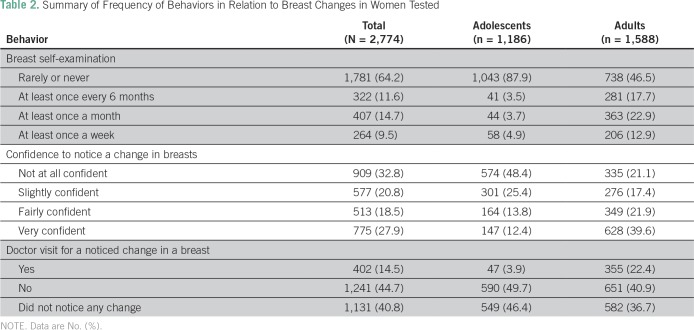
Summary of Frequency of Behaviors in Relation to Breast Changes in Women Tested

**Table 3:**
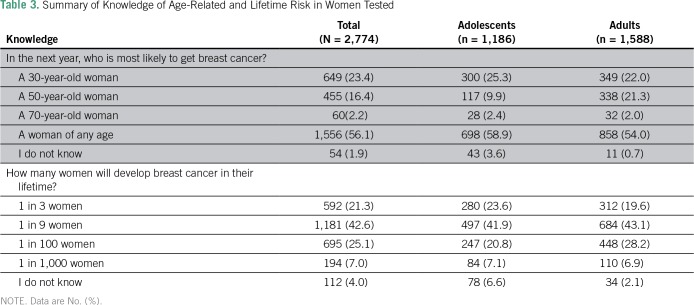
Summary of Knowledge of Age-Related and Lifetime Risk in Women Tested

A total of 1,675 participants (60.4%) knew about the availability of a BC screening program in Gaza. However, only 329 (23.8%) reported its true starting age at 40 years. In general, awareness of BC symptoms and warning signs as well as risk factors was low when open-ended (recall) questions were used and was higher with closed (recognition) questions. The overall mean ± SD score for recognition of BC symptoms was 5.9 ± 2.9 of 11, and adults demonstrated higher awareness than adolescents (6.8 *v* 4.8 of 11; *P* = .08). Adults demonstrated a significantly higher recall and recognition of all signs and symptoms of BC than adolescents except in recall about change of nipple position and change of breast/nipple shape (Table 4). A breast lump was the most commonly recognized symptom (n = 2,166; 78.1%), whereas pulling in of nipple was the least recognized (n = 1,009; 36.4%).

**Table 4:**
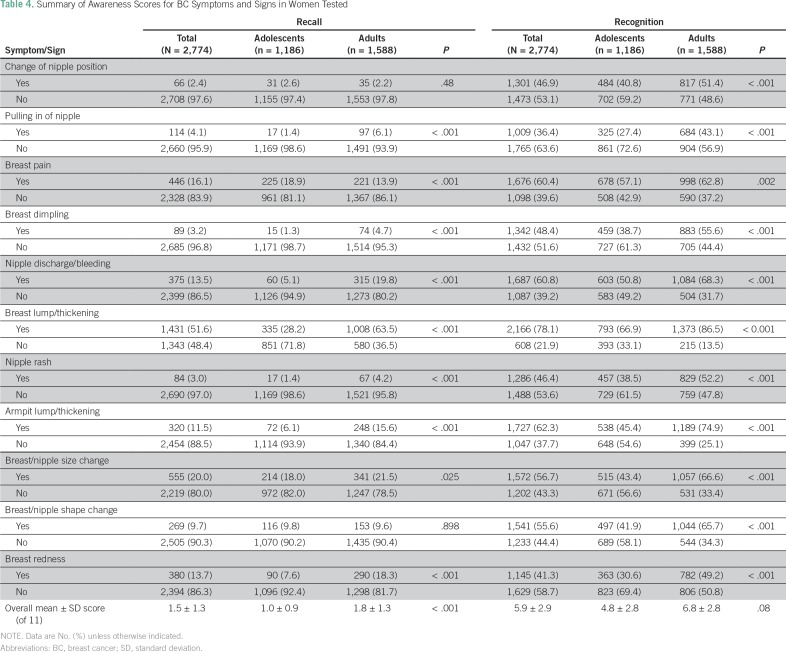
Summary of Awareness Scores for BC Symptoms and Signs in Women Tested

The overall mean ± SD score for recognition of BC risk factors was 7.5 ± 3.1 of 16, and adults showed a significantly better knowledge than adolescents overall (8.0 *v* 6.8 of 16; *P* < .001) as well as in every BC risk factor (Table 5). A past history of BC was the most frequently reported BC risk factor (n = 1,333; 48.1%) and early menarche was the least (n = 430; 15.5%).

**Table 5:**
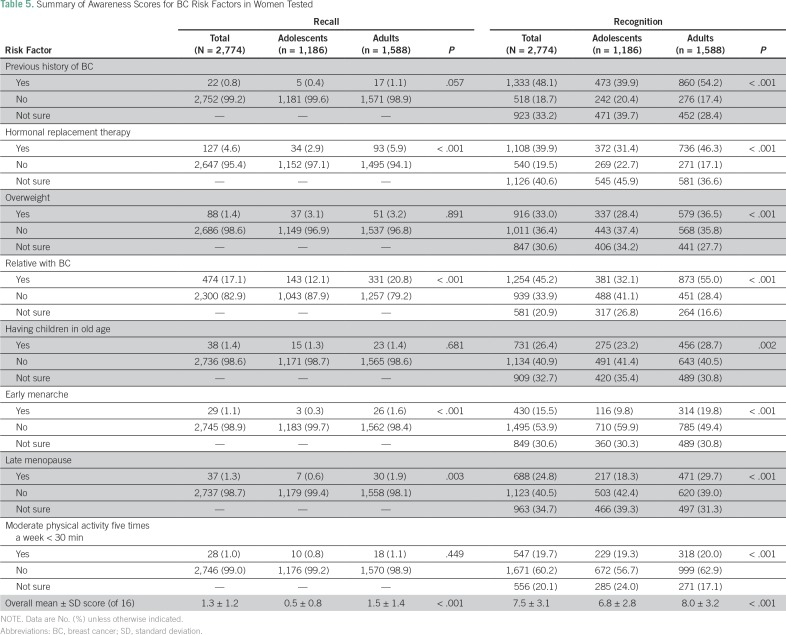
Summary of Awareness Scores for BC Risk Factors in Women Tested

Overall, emotional barriers were the most commonly reported barriers to seeking medical help; feeling embarrassed was the first reason (n = 1,536; 55.4%), and this was also the most frequently reported barrier among adolescents (n = 745; 62.8%). Conversely, feeling scared was the most frequently reported reason among adults (n = 802; 50.6%; Table 6).

**Table 6:**
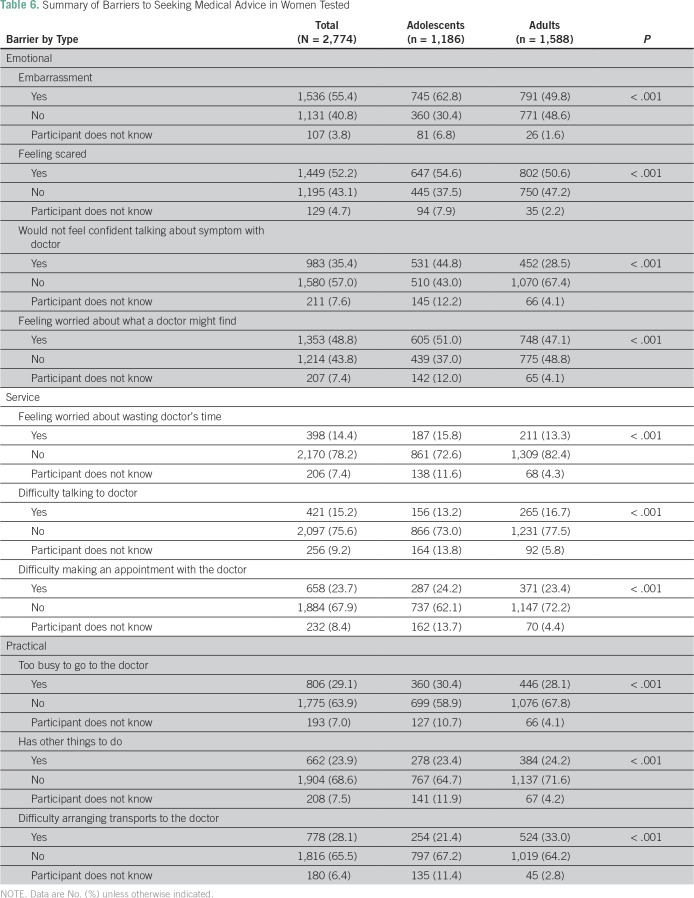
Summary of Barriers to Seeking Medical Advice in Women Tested

## DISCUSSION

The overall awareness of BC in this study was suboptimal. Adult women demonstrated higher awareness than adolescents, especially in observations about breast changes and knowledge about the age-related risk. A major difference was observed in the emotional barriers; feeling embarrassed was reported as the most common barrier to seeing a doctor by adolescents, and being scared was the most common barrier reported by adults.

To our knowledge, this is the first study to examine BC awareness in Gaza and Palestine, where—given the difficult economic circumstances and high prevalence of BC—effective prevention strategies and early recognition are essential to increase BC survival to international levels. Awareness of BC symptoms and risk factors as well as reduction of the barriers to seeking medical advice are crucial to achieve this goal. This study looked at these factors to support the development of effective policies in the public health and education sectors.

BSE is one of the most cost effective and accessible interval screening methods available.^7^ However, only 35.8% of participants reported BSE performance compared with 87.8% in a study from Bangladesh.^16^ Forbes et al^20^ found that 52.0% of women in East London were fairly or very confident in the ability to notice any breast change, and this rate was higher than the proportion found in this study—46.4%. In the same study,^20^ 23.0% checked their breast at least once a month compared with only 14.7% in this study. The congruent decrease of BSE performance and confidence in the ability to detect breast changes in this study could be explained by the fact that BSE increases body awareness, so there is heightened awareness of changes that may be detected if BSE is performed regularly.^21^ Reasons for the poor performance in BSE among Palestinian adolescents and women might be poor body awareness and lack of self-confidence to detect changes as well as the belief that a health care professional is needed for recognition of abnormalities. BSE is rarely talked about in the Gazan public, and findings of this study demonstrate the urgent need for public education of adolescents and women of all ages about BSE.

In contrast to this study, other studies showed better knowledge of age-related BC risk.^20,22^ This might reflect poor health education about the age-related risks of BC in the Gaza Strip. Similar to participants of studies conducted in India and Malaysia, participants in this study were able to recognize BC symptoms when asked closed questions.^23,24^ However, only a minority demonstrated accurate knowledge when asked open-ended (recall) questions, which reflects the greater difficulty of recall questions. In concordance with other studies from Kuwait and Jordan, breast lump or thickening was reported as the most frequently recognized BC symptom among women in this study.^25,26^ This result confirms the common belief that a breast lump is the most concerning BC symptom, from the participants’ perspectives, for which they will seek medical advice when recognized.

Interestingly, women in Gaza were more aware about the BC risk factors than those in the United States.^27^ It is thought that the social interactions and life in extended families, which are common in Gaza, may play roles in this discrepancy, because people can talk more with one another in person and share information from personal and family stories about BC. Another explanation could be the increased fear that participants in this study reported, which prevented them from presenting early to a doctor but also caused a heightened awareness of risk factors.

Older women had greater BC awareness than adolescents in the Gaza Strip. This was also observed in other studies in Qatar,^12^ Malaysia,^23^ and Bangladesh.^28^ A possible reason for this is that such issues are not part of the regular school curriculum; also, adolescent girls might not be interested in this issue. Adults, conversely, already had the opportunity to acquire knowledge from encountering similar problems over time or from reading and hearing from their friends or relatives. In addition, female adolescents possibly feel shy about reading or talking about BC or symptoms they might experience, which is in concordance with embarrassment as the most frequent barrier for presentation to a doctor in this study.

Awareness of the availability of a BC screening program in Gaza (60.4%) is slightly lower than that in Bangladesh (67.0%).^28^ Surprisingly, though, it is still higher than the awareness observed in East London, despite the fact that the UK breast screening program is highly organized and invites eligible patients via letter or doctors.^20^ Conversely, Gaza women rely entirely on their own motivation, initiative, and knowledge of the program to benefit from it. Therefore, a higher awareness would have been expected among women in East London. These unexpected findings might be caused by the high proportion of women in East London who come from an immigrant community and who may have poor language skills or poor knowledge of the local health system.

Forbes et al^20^ surveyed 1,515 participants in East London and reported a mixture of emotional and practical barriers most commonly encountered: 47.0% were worried about what the doctor might find, 38.0% were embarrassed to see the doctor, 37.0% were worried about wasting the doctor’s time, and 35.0% found it difficult to make an appointment. This is consistent with another study, in which practical barriers were most frequently reported.^29^ However, in this study, emotional barriers were the most commonly reported, and higher percentages in all emotional barriers versus physical or practical ones were obtained for each one. The poorer knowledge and awareness among Gaza women about BC, or local experiences with poor outcomes because of more advanced-stage presentation, may explain this variation. These reasons might drive emotional barriers (such as fear of diagnosis) higher in Gaza compared with other places.^20^ Another explanation could be that barriers for seeking medical help may depend on the presenting symptom. A previous study showed that women who experienced a breast lump were less likely to have a delayed presentation than those with other symptoms, like nipple discharge.^30^ Accordingly, Meechan et al^31^ found that although women with breast lumps waited a shorter time before seeking help, there was no difference in the level of emotional response to symptoms in the breast lump and non–breast-lump groups, which suggests that these factors may be operating independently. In contrast, breast lump was the most commonly reported BC symptom in this study, but most of the participants still reported emotional barriers that could potentially delay presentation. This is in concordance with data from Gaza, which showed that women with breast cancer are often diagnosed at more advanced disease stages because of delayed presentation.^32^ Another factor that may contribute to this delay is the embarrassment reported as a barrier to seeing a doctor by both adolescents and adults; embarrassment might be greater when faced with less well-recognized symptoms, such as nipple discharge. This factor might be potentiated by the lack of female surgeons in Gaza. The first female trainee only entered the surgical training in Gaza in 2016. Availability of female breast surgeons might reduce the embarrassment factor that leads to the delay in presentation.

The main strength of this study is the large sample that gives a representative view of the target population. Furthermore, the inclusion of women from different age groups and the high response rate show general acceptance of such a survey by the target population.

Limitations of this study include the lack of socioeconomic data and level of education, which can influence knowledge and awareness of BC, as well as the lack of additional exploration about how much influence factors, such as family history of BC and familiarity with the disease through friends and neighbors, had on the participants’ knowledge and awareness of BC.

The recruitment of women in hospitals might have led to the selection of participants who were more aware of health issues. Furthermore, the inclusion of visitors and the exclusion of oncology departments for recruitment reduced the number of participants with special awareness of BC because of illness of self or a close relative. An additional limitation was the lack of closer examination of the barriers to visiting a doctor earlier, because that question required a different study design.

Early BC detection and disease downstaging remain the cornerstones of BC control to improve its outcome and survival in low-income countries.^33^ This study provides evidence that the knowledge about BSE practice, symptoms and risk factors of BC, and availability of BC screening are low, which contributes to the advanced presentation and, hence, poor survival of patients with BC in the Gaza Strip. Educational interventions to raise public awareness of BC and to address the emotional barriers to presentation to a doctor, such as fear and embarrassment, are needed to encourage early presentation and improve outcomes.^29^ More female breast surgeons also must be available for consultation in the Gaza Strip to facilitate early presentation, especially of younger women.
